# Genome Sequences and Phylogeny of Two Duck Hepatitis B Viruses

**DOI:** 10.1128/MRA.01327-20

**Published:** 2021-02-18

**Authors:** Daniel Apolônio Silva de Oliveira, Yannick Debing, Ines Dieryck, Wilfred Michael Lyimu, Jan Paeshuyse

**Affiliations:** a KU Leuven, Department of Biosystems, Division of Animal and Human Health Engineering, Laboratory of Host-Pathogen Interactions in Livestock, Leuven, Belgium; b Aligos Belgium BV, Leuven, Belgium; Portland State University

## Abstract

Duck hepatitis B virus (DHBV) infection in Pekin ducks is a model for human hepatitis B. Sequence variations may contribute to host therapy responses against the virus. We provide full genome sequences of two DHBVs from France, their phylogenetic classification, and their sequence variability.

## ANNOUNCEMENT

Chronic hepatitis B, caused by hepatitis B virus (HBV), is a worldwide health burden currently affecting 260 million people and resulting in 2,000 HBV-related deaths per day (1). Duck hepatitis B virus (DHBV) belongs to the same family as HBV (*Hepadnaviridae*) but to another genus (*Avihepadnavirus*), with a structure very similar to that of HBV, i.e., an external envelope with duck hepatitis B surface antigen (DHBsAg) and a nucleocapsid surrounding partially double-stranded circular DNA. Both are noncytopathic viruses and can be vertically transmitted (2).

In April 2020, blood samples were randomly obtained from Pekin ducks (Anas platyrhynchos) that had been bought from a commercial duck breeder in western France and were housed at TRANSfarm, KU Leuven (Lovenjoel, Belgium). Two hundred microliters of blood was collected from the neck vein at 3 days of age. Here, we describe two different viruses named 410 and 414 based on their amplicons. DHBV DNA was extracted from serum using the DNeasy blood and tissue kit (Qiagen). Full genome amplification was performed with external primers P3 and P4 (3) (Table 1). PCR cycling conditions were initial denaturation at 94°C for 5 min, 32 cycles of denaturation at 94°C for 30 s, annealing at 57°C for 30 s, and extension at 72°C for 3 min 10 s, and final extension at 72°C for 7 min. For sequencing, internal primers D1 and D2 were designed using the Primer-BLAST tool (NCBI), and P5 and P6 primers (3) were used to ensure full genome coverage with the same PCR cycling conditions. All tools were run with default parameters unless otherwise specified.

**TABLE 1:** Primers used for amplification of the two viruses, 410 and 414

Primer^*a*^	Sequence	Direction
P3	AAT TAC ACC CCT CTC CTT CGG AG	5′ to 3′
P4	GTA ATT CTT AAG TTC CAC ATA GCC	3′ to 5′
P5	CAC CCC TCT CTC GAA AGC AAT A	5′ to 3′
P6	GAT AGT CAG GTT GAA AGC TCA C	3′ to 5′
D1	ACC ATA GAT CTC CCT CGC CT	5′ to 3′
D2	AAA AGA GCA GAC AGC GTG GC	3′ to 5′

a External primers P3 and P4 were used to amplify the full genome. Primers P5, P6, D1, and D2 were used to guarantee full genome coverage.

PCR products were purified using the Wizard SV gel and PCR clean-up system (Promega), and both strands were sequenced by classic Sanger sequencing (Eurofins). The sequence chromatograms from the genomic fragments were assembled using the standard consensus method and cutoff of the DNASTAR Lasergene SeqMan v. 7.1.0. package (trace evidence, evidence of 50%). Prior to phylogenetic analysis using the maximum likelihood method, a best-fit model analysis for nucleotide substitution was performed and the general time-reversible model with discrete gamma distribution (GTR+G) was chosen. We used the heuristic method with the initial tree being generated by standard neighbor-joining tree inference. The bootstrap consensus tree was set to 1,000 replicates. Other complete DHBV genome sequences were obtained from GenBank, and all analyses, including nucleotide and amino acid similarity analyses, were performed with the software package MEGA v. 10.1.7 (4).

**FIG 1:**
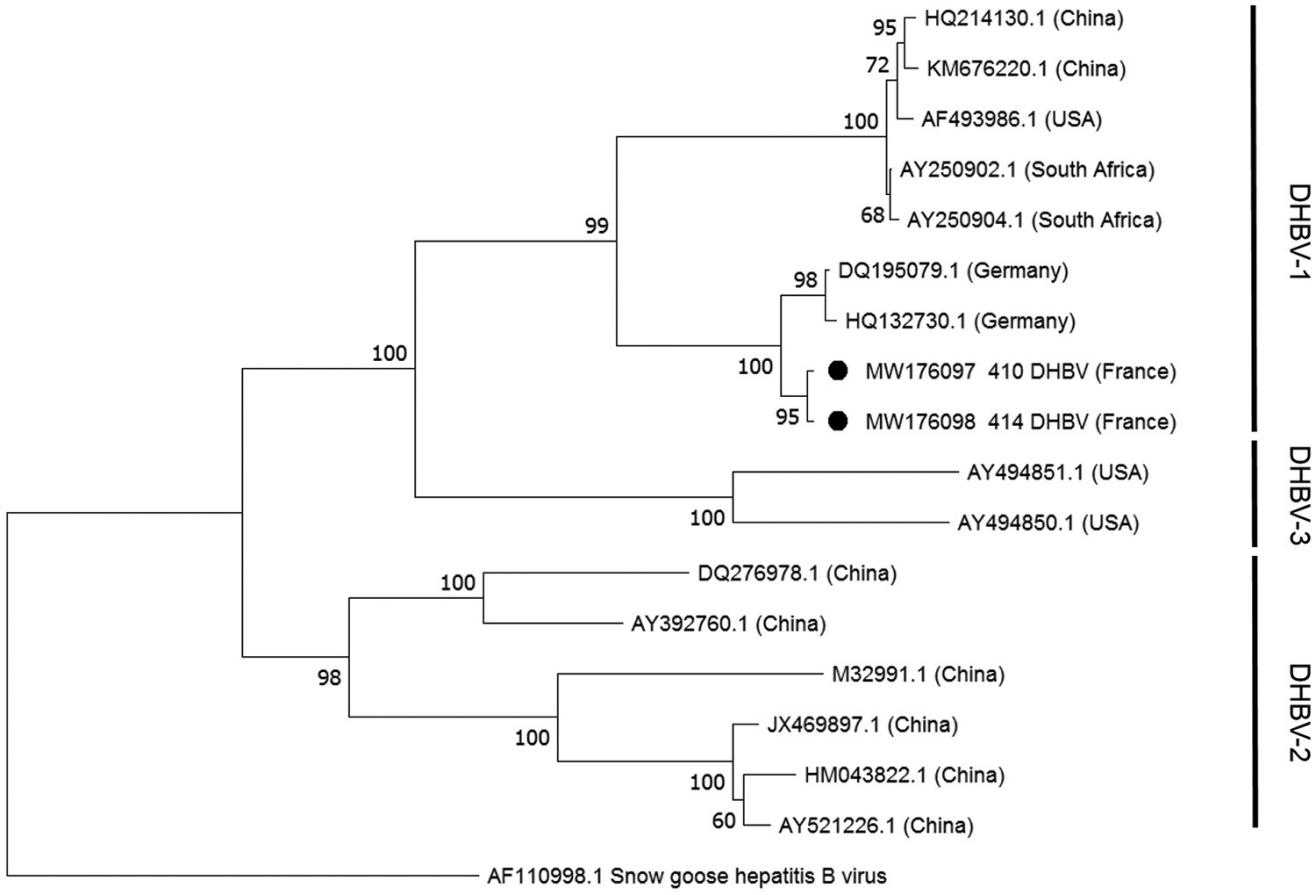
DHBVs 410 and 414 from France can be classified as the DHBV-1 genotype. The phylogenetic tree includes 15 DHBV complete genome sequences from GenBank, with their corresponding accession numbers. Our viral sequences are indicated by black dots. Bootstrap values of 1,000 tree replicates are shown at each node. Snow goose hepatitis B virus was chosen as the outgroup that rooted the tree.

DHBV is categorized in three distinct genotypes (5). Our two viruses had a full genome of 3,021 bp and grouped together with genotype DHBV-1 (Fig. 1). The two viruses showed 99.8% nucleotide sequence similarity and 99.6% amino acid sequence similarity. In the amino acid sequence, they differ in one amino acid in both pre-S and polymerase open reading frames (ORFs) (position 400 [bases 1198 to 1200]) and four amino acids in the terminal portion of the polymerase ORF and direct repeat 2 (DR2) region (position 811 [bases 2431 to 2433], position 824 [bases 2470 to 2472], position 825 [bases 2473 to 2475], and position 830 [bases 2488 to 2490]). The G+C contents were 43.1% and 43.2% for DHBVs 410 and 414, respectively, falling into the expected G+C content range for DHBVs (42.8% to 46.5%) (6).

### Data availability.

These whole-genome sequences have been deposited in GenBank under the accession numbers MW176097 (410) and MW176098 (414).
